# Vitamin D in Endocrine Disorders: A Broad Overview of Evidence in Musculoskeletal, Thyroid, Parathyroid, and Reproductive Disorders

**DOI:** 10.3390/ph19010054

**Published:** 2025-12-26

**Authors:** Balazs Lengyel, Richard Armos, Bence Bojtor, Andras Kiss, Balint Tobias, Henriett Piko, Anett Illes, Eszter Horvath, Zsuzsanna Putz, Istvan Takacs, Janos P. Kosa, Peter Lakatos

**Affiliations:** 1 Department of Medicine and Oncology, Faculty of Medicine, Semmelweis University, 1083 Budapest, Hungary; armos.richard@semmelweis.hu (R.A.); bojtor.bence@semmelweis.hu (B.B.); kiss.andras1@semmelweis.hu (A.K.); tobias.balint@semmelweis.hu (B.T.); piko.henriett@semmelweis.hu (H.P.); barkaszine.dr.illes.anett@semmelweis.hu (A.I.); g.horvath.eszter@semmelweis.hu (E.H.); putz.zsuzsanna@semmelweis.hu (Z.P.); takacs.istvan@semmelweis.hu (I.T.); kosa.janos@semmelweis.hu (J.P.K.); lakatos.peter@semmelweis.hu (P.L.); 2 Se Hun-Ren-Tki Endomolpat Research Group, 1085 Budapest, Hungary

**Keywords:** vitamin D, autoimmune thyroid disease, Hashimoto’s thyroiditis, Graves’ disease, postpartum thyroiditis, infertility, PCOS, endometriosis, osteoporosis, primary hyperparathyroidism

## Abstract

Vitamin D is well established for its skeletal effects, being a cornerstone of several endocrine disorders. In recent years, it has come under investigation as a potential disease-modifying drug in several endocrine disorders through its immune modulatory and anti-tumorigenic action, particularly in thyroid disease, gynecologic disorders, and general fertility. Vitamin D supplementation is well established in the treatment of osteoporosis, osteomalacia, hypoparathyroidism, and primary hyperparathyroidism. In autoimmune thyroid disease, there is a negative correlation between 25(OH)D3 levels and prevalence. Currently available data are inconclusive on supplementation as a disease-modifying treatment. In Hashimoto’s thyroiditis, while some found improved thyroid function, a decline in progression, and antibody titers, these findings were not consistent, and some found no improvements. Painless postpartum thyroiditis severely lacks evidence. Interventional studies failed to demonstrate benefits in Graves’ disease. The literature consistently reports lower vitamin D levels in infertility, polycystic ovarian syndrome (PCOS), and endometriosis. In PCOS, data suggest that vitamin D supplementation is beneficial; however, results in exact benefits vary and there is no consensus on dosing. Current guidelines support supplementation as part of preconception nutritional care. In general, for female infertility and endometriosis, the results are conflicting, with a lack of high-quality evidence. The literature suggests there is a possible benefit regarding sperm motility, but not in testosterone levels for males. In conclusion, while in vitro studies and animal models are promising, the available evidence is often contradictory, with high heterogeneity in study designs and populations. Our paper highlights the need for further high-quality research to resolve current controversies.

## 1. Introduction

Vitamin D3 supplementation is the cornerstone in the treatment of several endocrine disorders for its well-established role in skeletal and calcium homeostasis. It is a fat-soluble hormone primarily produced through de novo synthesis in the skin after ultraviolet radiation exposure. A secondary source of vitamin D is dietary intake from foods, such as salmon, eggs, and liver among others. Cholecalciferol itself requires hydroxylation by the liver (25-hydroxylation) and the proximal tubule of the kidneys (1-alpha-hydroxylation) into 1,25(OH)2 D3 to achieve its biologically active form. The body’s vitamin D stores are best reflected by the serum 25(OH)D3 levels [1]. In recent years, vitamin D has been extensively studied for its pleiotropic effects and potential role in aging, cardiovascular health, oncology, immunology, and gynecology. Since vitamin D is considered essential in several physiological processes through its genetic (e.g., through the vitamin D receptor [VDR]) and epigenetic effects (e.g., methylation) [1], vitamin D deficiency has been implicated in several diseases as a potential etiological factor. Several studies established a correlation between vitamin D deficiency and certain disorders, while establishing causation is the subject of rigorous research. This narrative review aims to provide an overview of well-established indications of vitamin D supplementation in endocrine disorders, while also summarizing its role and postulated therapeutic implications in reproduction and thyroid disorders through its proposed pleiotropic effects.

## 2. Methodology

For this narrative review, we searched for articles on PubMed, Google Scholar, Scopus, and Embase up until October of 2025. We collected relevant meta-analyses, systematic reviews, guidelines, and interventional and observational trials and strived to focus on articles with appropriate designs and novel results. We also included in vitro and animal studies, which demonstrated vitamin D’s pleiotropic biological effects. It is important to emphasize that our paper is a narrative review and, as such, it does not include all available studies and evidence.

## 3. Vitamin D Beyond Calcium Homeostasis: A Brief Overview of Its Potential Role in Immunomodulation and Oncogenesis

### 3.1. Vitamin D and the Immune System

Vitamin D influences inflammatory regulation via vitamin D receptor-dependent and -independent pathways, yet its effects on the innate and adaptive immune systems remain complex and incompletely elucidated. The genetic regulation of our body’s innate immune system is influenced by VDR-dependent action, particularly through monocytes, which are considered the most responsive to vitamin D. VDR regulates several genes that result in the activation of the LPS/TLR4 pathway, a key player in immediate response to bacteria and viruses [2]. Vitamin D is also known to downregulate Th1 and Th17 cells and stimulate regulatory T cells, the main reason why it is extensively studied for its potential therapeutic role in autoimmune disorders [3]. Through VDR, vitamin D modulates several genes (e.g., ACVRL1, CAMP, CD14, etc.) with key importance in the development of autoimmune disease [2]. Through paracrine action, it contributes to monocyte proliferation and differentiation, while reducing the overall inflammatory response of activated macrophages, suppressing pro-inflammatory (e.g., TNF-alfa) mediators and stimulating the production of anti-inflammatory mediators (e.g., IL10), thus balancing the inflammatory response [4]. Even though the comprehensive summary of the complex immune regulatory effects of vitamin D is beyond the scope of this paper, the aforementioned examples outline its potential role in the healthy function of our immune system and raises the question of whether vitamin D deficiency contributes to the development of autoimmune (e.g., Hashimoto’s thyroiditis, Graves’ disease) and infectious diseases.

### 3.2. Vitamin D and Cancer Development

Vitamin D has also been implicated in tumorigenesis, including colon, prostate, and breast cancer, amongst others. Primarily through the VDR, it affects both tumor cells and their microenvironment [5]. An important prerequisite for its antitumor action is the fact that most tumors retain VDR, with data suggesting that increased VDR receptors are in fact a good prognostic sign [1]. VDR signaling has been shown to cause cell cycle arrest enacted by transcriptional changes (e.g., downregulating cyclin-dependent kinases or upregulating their inhibitors such as p21), resulting in an antiproliferative effect [1,6,7]. On the other hand, it also contributes to apoptosis through shifting the balance of pro- and anti-apoptotic factors towards apoptosis, with a tumor-specific mechanism [1,6,8]. An anti-angiogenic and anti-metastatic action has also been demonstrated by the inhibition of vascular endothelial growth factor and transforming growth factor-β [9,10]. The previously described anti-inflammatory effects are also part of the anti-tumor effects by enhancing anti-tumor immunity and controlling chronic inflammatory processes [5,11,12]. These anti-tumor effects are still the subject of ongoing research, but warrant research into vitamin D’s potential role in the prevention and treatment of malignant disorders.

## 4. The Role of Vitamin D Supplementation in Osteoporosis and Health

### 4.1. The Basis of Vitamin D Supplementation in Osteoporosis

Osteoporosis is a population-wide disease characterized by low bone mass and pathologic microarchitecture resulting in an increased risk of fractures, most importantly hip fractures, leading to considerable morbidity, mortality, social burden, and healthcare spending [13]. The prevalence of osteoporosis worldwide is estimated to be around 21.7% amongst people aged 50–85, with it being three times more common in women [14,15]. The basis of diagnosis is bone densitometry with a DEXA scan, with a diagnostic threshold of −2.5 standard deviations. Fracture risk is calculated using FRAX, which incorporates several risk factors besides bone mineral density (BMD) to assess ten-year fracture risk, highlighting the multifactorial considerations when treating osteoporosis [13]. Vitamin D deficiency is a major risk factor for osteoporosis [16] and is considered a cornerstone of nutritional interventions, combined with calcium supplementation. Vitamin D deficiency is proposed to result in impaired intestinal calcium absorption, and the resulting hypocalcemia tendency leads to secondary hyperparathyroidism, which in turn will promote bone resorption [17].

The International Osteoporosis Foundation’s guideline recommends an intake of 800 IU of vitamin D and a calcium intake of 800–1000 mg per day for everybody above 50 to provide appropriate nutrition for skeletal health. An intake of 400–800 IU of vitamin D and 500–1200 mg of calcium supplements is recommended for every patient receiving osteoporosis therapy, since most studies for bone protective interventions were designed with co-administration of both [18]. In fact, studies point to vitamin D repletion being a prerequisite for appropriate bisphosphonate response and improved efficacy [19,20,21,22,23,24]. In presumed bisphosphonate non-responders, vitamin D repletion to levels ≥ 33 ng/mL significantly restored the therapeutic response to bisphosphonates [21]. Compared with alendronate monotherapy, combined alendronate and vitamin D treatment was associated with superior improvements in bone mineral density, bone turnover parameters, and normalization of vitamin D status in a randomized controlled trial [22]. Meanwhile, others demonstrated that the combination therapy was also superior in suppressing parathyroid hormone (PTH) spiking after bisphosphonate therapy [23]. It is also important to highlight that studies which compared vitamin D and calcium alone vs. in combination suggested that only the combination is likely to decrease fracture risk [25,26]. In summary, vitamin D deficiency can severely limit the efficacy of antiresorptive therapies and thus must be addressed in every patient [21,22,23,24].

The most contested issue in the field is the optimal dosing of vitamin D regarding daily vs. weekly, and the optimal amount per month. Several large meta-analyses found that vitamin D supplementation decreases fracture risk with an optimal dose around 800–1000 IU/day, with diminishing results at lower (<400 IU) and potentially detrimental results at higher doses [27,28]. Particularly higher doses are associated with a higher risk of falls and fractures [29,30]. The mechanism for the latter is thought to be a result of decreased muscle function and abnormal bone turnover and mineralization, suggesting a U-shaped effect [31,32]. Due to this concern, certain guidelines show a preference toward daily/weekly administration, discouraging monthly supplementation, while others found the regimes comparable in efficacy and safety, showing no clear preference [33,34].

On the other hand, we have to highlight that others found vitamin D supplementation an ineffective measure to reduce fracture risk, such as Lai et al. [35,36] in a meta-analysis of seven RCTs demonstrating no statistically significant improvement in hip fracture risk compared to placebo, while also acknowledging the studies’ limitations and the several potential factors that might significantly influence results [35]. Nonetheless, current expert consensus guidelines support the use of vitamin D supplements combined with calcium in patients at high risk of fractures, receiving anti-resorptive therapy and suffering from deficiencies, while emphasizing the need for further high-quality research to establish correct doses, dose intervals, and indications for supplementation [13,18].

### 4.2. Preventive Supplementation

Another important question is whether vitamin D supplementation is beneficial as a preventative measure in healthy individuals. Large RCTs such as the VITAL trial failed to demonstrate a decrease in fracture risk when supplementation was used in the general population [37,38]. Furthermore, due to uncertain threshold and considerable cost of 25(OH)D3 measurements, current international and local guidelines advise against routine screening, while also highlighting the need to consider special at-risk populations with higher demand or lower intake/synthesis of vitamin D (e.g., childhood, pregnancy, lactation, chronic kidney failure, malabsorption syndromes, darker complexion, old age > 75, and obesity). The approach differs between guidelines when considering whether to test at-risk populations or only administer routine amounts of vitamin D [33,39,40].

For example, in the Hungarian guideline, it is highlighted that since the population receives little to no UV radiation during the months from November to March, every adult should receive supplementation, which takes into consideration the unique weather conditions for the country, emphasizing the importance of individualizing recommendations for the local geographical and cultural landscape [33].

## 5. Are Patients with Primary Hyperparathyroidism Truly Vitamin D Deficient?

Epidemiological data suggest that vitamin D insufficiency (<30 ng/mL) is present in 18–36% of patients with primary hyperparathyroidism (PHPT) with 9–12% of the cases having severe vitamin D deficiency (<10 ng/mL) [41,42,43]. This is significantly higher than in the matched healthy population. Some suggested that secondary hyperparathyroidism due to vitamin D deficiency might be a potential cause of PHPT as a result of consistent stimulation of parathyroid cells, due to low calcium and vitamin D levels. Furthermore, according to Gillis et al. [44], vitamin D deficiency is associated with single gland disease, which might support the hypothesis that a singular adenoma could ensue from constant stimulation of otherwise healthy parathyroid cells by vitamin D deficiency and its consequences. Nonetheless, the current expert consensus does not support this statement, pointing to the fact that the incidence of PHPT increased, even though the 25(OH)D3 levels of PHPT patients improved in recent years [44,45].

While vitamin D deficiency seems to be common in PHPT, it is important to highlight that high levels of PTH stimulate 1-alfa-hydroxylation of 25(OH)D3 into 1,25(OH)2 D3. The increased conversion might be a minor contributor to hypercalcemia. Levels of 1,25(OH)2 D3 are not routinely measured; thus, the literature suggests the increased conversion might be in part the cause of low 25(OH)D3 levels. Further supported by the fact that post-parathyroidectomy 25(OH)D3 levels show considerable improvement [46,47,48]. On the other hand, if somebody is truly vitamin D deficient, the decreased intake and de novo synthesis combined with increased turnover can result in the consumption of stored vitamin D, as 25(OH)D3 has a half-life of 1–2 weeks, while 1,25(OH)2 D3 only has a half-life of 4–14 h [49,50,51]. In turn, lack of vitamin D stimulates further PTH secretion [52].

Another consideration is that while the total 25(OH)D3 is diminished, the free fraction does not seem to be abnormal [53,54]. Considering the previous statements, the question may arise as to why it is important to replete vitamin D in PHPT patients, if there might not be a frank deficiency in at least a part of the cases. In fact, current international guidelines recommend screening in all patients and maintaining 25(OH)D3 levels between 30–50 ng/mL [55].

The rationale behind this recommendation is that low 25(OH)D3 levels result in a more severe biochemical profile with a higher incidence of complications. Vitamin D deficiency is inversely correlated to gland size, serum PTH, alkaline phosphatase, and calcium levels [41,56,57,58]. The impact of vitamin D deficiency on bone health seems to be moderate based on current evidence, mostly affecting the cortical bone [41,59].

There are concerns over the danger of vitamin D supplementation exacerbating hypercalcemia. However, these fears are unfounded, as multiple studies have demonstrated that with appropriate monitoring, vitamin D repletion can safely lower PTH levels and bone turnover parameters [52,58]. Overall, vitamin D supplementation in PHPT is an important, safe, and well-established intervention in every patient with vitamin D insufficiency and deficiency. Still, it is important to point out that the exact role of it in PHPT is yet to be elucidated and should be the subject of further research.

## 6. Hypoparathyroidism

Hypoparathyroidism is a rare disease with a prevalence of around 30/100,000/year [60,61]. The disorder is defined by the lack of PTH, which can be a result of variable etiologies [61], the most common of which is postsurgical iatrogenic hypoparathyroidism [61,62]. Other causes include autoimmune destruction, genetic causes such as DiGeorge syndrome, infiltrative disorders, radiation damage, and hypomagnesemia [61,62]. Major pathophysiological features are hypocalcemia and hyperphosphatemia, the latter resulting from excessive renal reabsorption, which is normally inhibited by PTH [61,63,64,65]. Hypocalcemia results from decreased renal tubular reabsorption, decreased bone resorption, and decreased intestinal absorption due to a lack of 1-alpha-hydroxylation in the kidneys, resulting in low 1,25(OH)D3 levels [61,63,64]. The clinical picture is dominated by the signs and symptoms of hypocalcemia and hyperphosphatemia-driven extra skeletal calcification with complications such as cataracts, nephrocalcinosis, nephrolithiasis, vascular calcification, and basal ganglia calcification, amongst others [61,62]. The diagnosis is based on low calcium levels, in the presence of hyperphosphatemia and inappropriately low or normal PTH levels [61,66].

The abnormally low bone turnover resulting from PTH deficiency leads to an increased BMD; however, it also results in abnormal microarchitectural changes [67,68]. The BMD increment is usually the most pronounced at the lumbar spine. Cortical porosity and thickness have been demonstrated to be reduced on high-resolution peripheral quantitative computed tomography at the tibia and radius [69,70], while others describe an increased cortical thickness on bone biopsies [67]. Trabecular bone volume and the number of trabeculae are increased; on the other hand, the thickness of trabeculae and trabecular separation are decreased [69,70,71]. Trabecular bone score is normal or modestly decreased according to current evidence, pointing to the fact that increased BMD does not equate to improved trabecular microarchitecture, nor improved bone strength [69,70,72]. Overall bone strength seems to be comparable to healthy controls [69]. Available data regarding fracture risk is limited. However, according to a current meta-analysis by Pal et al., vertebral fractures seem to be significantly increased, especially in non-surgical cases; on the other hand, there is some data available suggesting a decreased femur fracture risk [73,74]. The exact osteological outcomes of hypoparathyroidism remain to be determined.

The cornerstone in the management of hypoparathyroidism is vitamin D and calcium supplementation. Other therapeutic options include magnesium supplementation when deficient, PTH analogue therapy, hypercalciuria control with thiazides, and hyperphosphatemia management with dietary restrictions and binders [61,75]. The main goal of therapy is to keep calcium levels at the lower end of normal or just below the normal range, to prevent symptomatic hypocalcemia while striving to minimize hypercalciuria and nephrocalcinosis. Vitamin D therapy can also exacerbate hyperphosphatemia brought on by increased intestinal phosphate absorption [61,68,75,76]. An important question of the field is the exact role of active versus inactive vitamin D supplements. Current guidelines favor active vitamin D forms such as Calcitriol, since they circumvent the lack of 1-alpha-hydroxylation and have a short half-life, which in turn allows for precise titration and makes it easier to prevent iatrogenic hypercalcemia [60,68,75]. Currently, inactive forms such as cholecalciferol have a role as an adjunct. The rationale for their use is to provide sufficient amounts of substrate for any residual 1α-hydroxylase activity, to support endogenous active vitamin D production [61,75,76], which in turn might lead to less fluctuation in calcium levels owing to their longer half-life and decrease the incidence of symptomatic hypocalcemia, especially in those patients who experience them despite Calcitriol therapy [68,75,76,77], with the caveat that toxicity can result in persistent hypercalcemia, thus requiring monitoring [61]. Current guidelines recommend keeping 25(OH)D3 levels between 30–50 ng/mL [76]. Data regarding vitamin D status is lacking. The potential systematic benefits of inactive vitamin D supplementation remain a subject of active research [78].

## 7. Vitamin D Deficiency and Osteomalacia

Unlike several other conditions discussed in this paper, causality is clearly established in osteomalacia, where vitamin D deficiency is the predominant etiological factor. The disease is characterized by the abnormal mineralization of the osteoid. The main pathophysiological process behind the disease is either the lack of adequate amounts of calcium and phosphate, decreased alkaline phosphatase activity, or inhibitors of mineralization such as aluminum and iron supplements. Major etiological factors include vitamin D deficiency due to nutritional causes or malabsorption, lack of solar exposure, disorders of vitamin D metabolism and action, drug or heavy metal exposure-induced forms, and hypophosphatemia forms of so-called hereditary fibroblast growth factor 23 (FGF23) mediated hypophosphatemic disorders [79,80,81]. A rare manifestation of the disease is tumor-induced osteomalacia. It is a result of ectopic FGF23 production that leads to renal phosphate wasting and the suppression of 1-alpha-hydroxylase in the proximal tubules, resulting in hypophosphatemia and reduced calcitriol synthesis, exacerbating the condition by further impairing intestinal calcium and phosphate absorption [82,83,84].

The above-mentioned factors clearly denote that osteomalacia is not a single disease entity, rather an umbrella term for multiple disorders [80]. Due to nonspecific clinical signs and symptoms (bone pain, muscle weakness, fatigue, and fractures) and the lack of reliable imaging signs, the diagnosis is based on a high index of suspicion and biochemical findings [80]. Thus, the disease is severely underdiagnosed with a known prevalence of only 0.1–0.4% in the general population [85,86]. Autopsies revealed osteomalacia in 20–36% of the cases [87,88]. Furthermore, if misdiagnosed as osteoporosis, antiresorptive therapy further decreases bone mineralization and may worsen the already present hypocalcemia [89,90].

Therapy should be directed at the underlying deficiency. In the case of vitamin D deficiency, the American Association of Clinical Endocrinologists recommends an initial loading dose of 5000 IU for 8–12 weeks followed by a maintenance dose of 1000–2000 IU combined with 1000–1200 mg of calcium either as dietary intake or supplements [91,92]. In hypophosphatemic cases, therapy should consist of oral phosphate supplements combined with active calcitriol supplementation [93]. The above-discussed data call for more clinical awareness regarding osteomalacia, especially considering the potentially catastrophic consequences of mistreatment with antiresorptive agents.

## 8. Vitamin D and the Thyroid

### 8.1. Hashimoto’s Thyroiditis and Vitamin D

Hashimoto thyroiditis/autoimmune thyroiditis (HT) is the most common etiological factor of hypothyroidism in developed countries, predominantly affecting female patients [94]. While the etiology of HT is proposed to result from genetic predisposition, including VDR polymorphisms and environmental triggers such as infections, stress, drug exposure, amongst many others, the exact mechanism is not well established [95]. But the disease ultimately result in the loss of self-tolerance, lymphocytic infiltration of the thyroid gland, T cell-mediated thyroid destruction, and the production of antibodies such as anti-thyroid peroxidase antibody (ATPO) and anti-thyroglobulin antibody (ATG), exacerbating the process [96,97,98].

It is important to mention that a significant portion of the population exhibits ATPO positivity and radio morphological abnormalities on ultrasound without clinically manifest disease, present within around 20% of the population [99]. This so-called euthyroid autoimmune thyroiditis has a transformation rate of 2–4%/year into overt hypothyroidism [100].

Since the current cornerstone of therapy is levothyroxine supplementation for life, other disease-modifying therapeutic options to prevent progression or reverse hypothyroidism have been highly studied in recent years. One such potential option that was the subject of rigorous research is vitamin D supplementation in HT patients with vitamin D deficiency. Vitamin D’s postulated immunomodulatory action is pleotropic. Several potential mechanisms have been described, such as downregulating the HLA II gene, restoring regulatory T-cell function, modulating B cell response, and decreasing antigen-presenting cell-driven T-cell activation [101].

Several observational studies and meta-analyses found that there is an inverse correlation between HT and serum 25(OH)D levels. Štefanić et al. [102] found in a meta-analysis that included 2695 HT and 2263 control patients that HT patients had a 3.21 odds ratio of having vitamin D deficiency. Other larger meta-analyses also support these claims [103]. There are several observational studies reporting a decline in ATPO and ATG levels as a result of vitamin D supplementation. In an interventional trial, Mazokopakis et al. [104] found a 20.3% decrease in ATPO levels after 4 months of supplementation. There is evidence that vitamin D supplementation reduces the risk of progression to overt hypothyroidism and improves thyroid function [105]. Some authors found improvements only in TSH and ATG but no changes in ATPO levels [106].

While these findings, amongst many others, are promising, it is important to highlight that the field lacks large RCTs; most of the studies are observational with significant heterogeneity in study populations, supplementation doses, and outcomes are inconsistent. Several studies found no significant improvement with vitamin D supplementation in any of the relevant parameters regarding HT [107,108,109], such as a retrospective study by Karakaya et al. [110], which included 81,180 patients and found no significant association between vitamin D and HT.

Based on current evidence, no recommendation can be given on vitamin D’s role in the management of HT patients as a disease-modifying option as its role remains controversial. Nonetheless, it is important to highlight that there is a large volume of evidence, regardless of disease-modifying effects, that vitamin D deficiency is common amongst HT patients and thus screening for it and initiating supplementation to prevent known complications of deficiency might be rational. To find a definite answer to the exact role of vitamin D’s immunomodulatory effects in HT, further large prospective RCTs are required.

### 8.2. Postpartum Thyroiditis and Vitamin D

During pregnancy, the body’s immune system is attenuated to allow maternal fetal tolerance, and results in the transient improvement in some autoimmune disorders, mainly the Th1 helper cell-driven ones, such as multiple sclerosis, rheumatoid arthritis, and autoimmune thyroiditis, which then tend to rebound after delivery [111,112,113]. This change is mainly explained by a shift from Th1 to Th2 dominance; in fact, diseases that are Th2 dependent, such as systemic lupus erythematosus, tend to flare up during pregnancy, which highlights that rather than immunosuppression, there is complex immunomodulation to accommodate this unique physiological state [112].

One such disorder is the painless postpartum thyroiditis (PPT), defined as a lymphocytic, often transient destruction of the thyroid tissue that develops within a year after pregnancy [114]. While its pathophysiological bases are similar to Hashimoto thyroiditis, it is distinct not only by its onset but also by histological differences, namely the lack of germinal centers or fibrosis and a generally more favorable outcome [112,115]. PPT has a prevalence of 4.4–5.7%, mostly affecting women with positive ATPO in the first trimester or after delivery, with about one fourth to a half of all patients developing permanent hypothyroidism long term [112,116,117].

With robust evidence for the link between Hashimoto thyroiditis and vitamin D status, one would assume that PPT would also be linked with vitamin D metabolism. As of now, three studies were conducted regarding vitamin D status and PPT, with only one of them on vitamin D supplementation as a potential intervention [118,119,120]. Krysiak et al. [118] in an observational pilot study involving a total of 59 patients, investigated the differences in 25(OH)D3 and PTH levels in hypothyroid, euthyroid PPT patients, patients with non-autoimmune hypothyroidism and healthy control patients for six months with standardized L-thyroxine treatment in the hypothyroid groups. They found a clear correlation between PPT and low 25(OH)D3 levels, being the lowest in PPT patients, aligning with previous findings in HT. PTH levels were also considerably increased compared to non-autoimmune hypothyroid participants. Furthermore, they found that L-thyroxine therapy increased 25(OH)D3, and reduced PTH levels only in PPT, which might be attributed to the anti-inflammatory and thyroid cell stabilizing effects of L-thyroxine supplementation previously described in Hashimoto’s thyroiditis [118].

In a follow-up interventional control matched study involving 38 women with L-thyroxine-treated PPT, the researchers investigated the effects of vitamin D supplementation on antibody titers and thyroid hormone levels after 3 months of therapy. Vitamin D supplementation of PPT patients resulted in lower ATPO levels than in the control group, suggesting that it may have an additive benefit to L-thyroxine therapy’s immunomodulatory effects, which may prove particularly beneficial, considering that higher ATPO levels are associated with developing permanent hypothyroidism [120]. Lastly, in a recent observational study comparing PPT patients with hypothyroidism, grouping patients based on vitamin D status, the authors described a considerable loss of the beneficial effects of L-thyroxine supplementation on female sexual health in vitamin D-deficient subjects [119,121]. This finding implicates that T4 requires vitamin D to exert its full effects or might implicate that vitamin D is an independent key factor for female sexual health [119].

The limited data available reflect the considerable difficulties of conducting interventional studies in an obstetric population, especially due to ethical and safety concerns. While we can hardly draw any far-reaching conclusions from these pilot trial results, PPT affects a large portion of childbearing women and its link to vitamin D, especially the potential beneficial effects of the supplementation, might prove to be a beneficial additive therapy to L-thyroxine supplementation in the future.

### 8.3. Basedow-Graves’ Disease and Vitamin D

Basedow–Graves’ disease (BGD) is the most common cause of hyperthyroidism worldwide [122,123]. The underlying pathophysiology is characterized by the production of an antibody with an agonistic effect on the TSH receptor, fibroblasts, and adipocytes, resulting in hyperthyroidism and the typical extrathyroidal manifestation of endocrine orbitopathy and pretibial myxedema. The latter two are the results of retro-orbital inflammation with glycosaminoglycan build-up and disruption of orbital muscles and infiltration of the dermis with glycosaminoglycans, respectively.

Only a fraction of patients present with the classical clinical triad. In fact, many of the cases are accidental findings on routine laboratory screening with only mild symptoms. On the other hand, thyroid storm, the severe manifestation of BGD, is a life-threatening condition [124]. The susceptibility to develop BGD has a major genetic component with a large number of immunoregulatory genes implicated, such as HLA, CTLA4, and CD4 [125].

The disease is ten times more predominant in women. Many environmental triggers have been identified, such as stress, the postpartum period, infections, iodine, and radiation exposure [124]. The diagnosis is established by evidence of hyperthyroidism and the detection of TSH receptor antibodies (TRAb). Conservative management is based on the use of thionamides, such as methimazole or propylthiouracil. While very effective at treating hyperthyroidism, they have the considerable drawback of significant adverse effects, namely, bone marrow suppression and hepatotoxicity. If a patient develops these conditions, medical management can become difficult, although lithium, potassium iodine, and beta-blockers may be used as alternatives; however, they lack the efficacy of thionamides [126,127].

The disease may resolve after 12 months of conservative therapy, but it relapses in about 54% of cases, especially in cases where TSH remains suppressed and TRAb levels are high despite therapy [124,128]. In these cases, definitive treatment is required by surgery or radioiodine therapy, which leads to hypothyroidism in the majority of cases [124].

BGD can lead to severe complications and significantly impact the patient’s quality of life, calling for improved medical therapy. One such effort to broaden the available arsenal of medications was the study of vitamin D as a potential adjunct to established therapies, with potential immunomodulatory effects. From observational studies, it is well established that BGD is associated with lower serum 25(OH)D3 concentrations, like in other autoimmune thyroid diseases [103,129]. Furthermore, it has been suggested that vitamin D deficiency is a risk factor for developing BGD. According to extensive evidence, vitamin D deficiency is present in roughly 40% of Japanese patients with benign gastrointestinal disease [130,131,132].

The proposed mechanisms previously discussed are linked to the immunomodulatory effects of vitamin D, supposedly contributing to the loss of immune tolerance [101]. The field is not extensively studied. There is some data suggesting a beneficial effect of vitamin supplementation [133,134]. However, medium to large-sized interventional trials failed to demonstrate significant improvements in clinical outcomes [135,136,137,138,139]. Most importantly, in the DAGMAR trial with an intervention period of 2 years and a follow-up period of 1 year, they failed to demonstrate any benefit from vitamin D supplementation in terms of recurrence of BGD regardless of vitamin D status [135]. They reported no improvements in TRAb titers neither thyroid function [135].

There is available data from another large trial involving 210 patients with the same follow-up period, that vitamin D supplementation might postpone recurrence, but overall recurrence rates remained similar [140]. These latter results may point to the fact that vitamin D is unlikely to significantly influence long-term autoimmunity in BGD. There were no studies available with longer post-intervention follow-up periods. While most of the recurrence occurs within the first 2 years of therapy discontinuation, the relatively short follow-up might obscure potential beneficial outcomes [124]. A medium-sized RCT in Egypt reported a statistically significant improvement in thyroid function, exophthalmos and thyroid volume [134]. With the caveat that the follow-up period was only 3 months, severely limiting the significance of these findings in BGD [134].

The other outlier by Gallo et al. [141] investigated the effects of combined selenium and vitamin D therapy compared to just methimazole, with a follow-up period of 270 days. Thyroid function and quality of life were significantly improved, which may suggest either that the selenium alone was responsible for the differences or that there is a synergistic action between vitamin D and selenium. However, the reduction of TRAb levels did not differ between the two groups [141]. In conclusion, based on current evidence, vitamin D supplementation as a disease-modifying therapy in BGD is currently not supported.

### 8.4. Thyroid Cancer and Vitamin D

Differentiated thyroid cancer is on the rise worldwide; however, it is questionable whether this is a result of a true rise in cases or a consequence of the widespread availability of modern diagnostic modalities resulting over-detection of early cases, demonstrated by the fact that mortality remains unchanged [142,143,144]. Nevertheless, advanced aggressive thyroid cancers remain an unsolved issue with anaplastic thyroid cancer having a two-year survival of 11% requiring further research for new therapeutic and preventative options [145,146].

An association between thyroid cancer and vitamin D deficiency has been described by in vitro studies, animal models, several observational studies, case studies, and meta-analyses. In in vitro thyroid cancer cell lines, vitamin D has been shown to decrease cell migration in a dose-dependent manner [147] while also inhibiting the secretion of CLL2 and CXCL8, two pro-tumorigenic chemokines [147]. Others also found cell growth inhibiting effects [148]. In a mouse model of follicular thyroid cancer, significant tumor volume reduction linked to increased levels of p27, a cyclin-dependent kinase inhibitor, was found [149]. A recent meta-analysis by Yang et al. [150] reported that low vitamin D levels were associated with the risk of thyroid cancer. However, the data were limited due to a lack of standardization amongst included studies [150]. On the contrary, both a large prospective observational trial of benign vs. malignant thyroidectomies and a retrospective study of 433 thyroidectomy cases failed to demonstrate a significant difference in vitamin D levels [151,152].

While the evidence from in vitro and animal models is compelling, the quality of available clinical data is low, and studies are sparse. As of now, no RCTs have investigated the pleiotropic effects of vitamin D in the context of thyroid cancer treatment or prevention, highlighting a critical area for future research.

## 9. Vitamin D’s Role in Fertility

### 9.1. Vitamin D and Infertility in General

Infertility is a major issue affecting couples worldwide, with a prevalence of 8–12% in European couples, and the trend is increasing in recent years [153,154,155]. From these, the male partner is responsible solely in 20–30% of the cases, and contributes in at least some part in 50% of the cases [154]. The economic impact is immense, with France alone spending EUR 70 million/10,000 women aged between 18–50 on infertility care [156]. Elucidating any potentially modifiable etiological factor is of paramount importance. One such factor that might play a role in fertility is vitamin D, supported by the fact that VDR and enzymes that metabolize vitamin D are highly expressed in male and female reproductive organs, and several animal models demonstrated its influence on reproductive physiology, such as steroidogenesis, sperm quality, and menstrual cycle regularity, amongst others [157,158].

### 9.2. Vitamin D in Female Infertility

The proposed role of vitamin D in female fertility is multifaceted. Follicle maturation is influenced through VDR-dependent action, impacting theca and granulosa cell hormone production and receptor expression, also modulating progesterone metabolism and anti–Müllerian hormone (AMH) signaling. In oocyte maturation and function, calcium signaling is crucial, which in turn is majorly impacted by vitamin D [159]. In animal models, vitamin D has been clearly shown to influence steroid production, demonstrated by vitamin D null mice having decreased aromatase activity amenable by vitamin D repletion [157].

Current research suggests that vitamin D deficiency is associated with unfavorable outcomes, including lower live birth rates and diminished follicular reserves [160,161,162]. The proposed immunomodulatory, anti-inflammatory, and antioxidant effects also contribute to implantation and pregnancy maintenance [162]. Anti-Müllerian hormone level is a marker of follicular reserves. Vitamin D levels are correlated with AMH, seasonal fluctuations paralleled by vitamin D levels, and also a sharp increase in AMH levels with acute supplementation of vitamin D [163]. Kuroshli et al. [164] demonstrated that it upregulates key implantation-related genes through VDR-dependent action, potentially improving endometrial receptivity and decidualization. They concluded that vitamin D repletion could improve implantation rates in patients with recurrent implantation failure [164].

Current epidemiologic data suggest that infertile women have vitamin D deficiency in 27% to over 80% of the cases, with vitamin D deficiency being associated with decreased fertility [165,166,167].

The available data on the exact clinical benefits of vitamin D supplementation remain a subject of debate. In a large meta-analysis including 2352 women from 12 studies, Meng et al. [168] reported that moderate dose (1000–10,000 IU/day) vitamin D supplementation for 1–3 months significantly improved clinical pregnancy rates, with a particular benefit in vitamin D-deficient patients. They found no benefit in miscarriages or biochemical pregnancies. However, we have to emphasize that the included studies were small, with considerable heterogeneity regarding design, study population, duration, and a lack of post-intervention vitamin D level control in all but three of the included studies [168].

Others also reported in an umbrella review that vitamin D raised clinical pregnancy rates in PCOS/IVF patients, although the certainty of evidence was rated very low, and the review also failed to demonstrate any improvements in other pregnancy-related parameters [169]. Zhou et al. [170] only reported improvements in biochemical pregnancies from five RCTs, with no benefits in clinical pregnancy rate, embryo quality, or miscarriage rate. However, the evidence level was limited by considerable heterogeneity, which is a limitation of all available meta-analyses [170]. The SUNDRO trial [171] investigated the effects of vitamin D supplementation in 630 patients with low vitamin D partaking in IVF in a two-center randomized double-blinded placebo-control trial. They concluded that vitamin D supplementation did not improve clinical pregnancy rates, nor secondary outcomes such as embryo quality, miscarriages, or birthweight [171]. Despite the study being well designed, we have to mention that they used a single-dose strategy (600,000 IU vitamin D), while others found beneficial effects with moderate doses, which in turn may point to the fact that vitamin D has been described to have a U-shaped effect in other settings [169,171].

In conclusion, while vitamin D has strong biological bases in relation to fertility, and observational studies consistently report lower vitamin D levels in infertile women and some evidence suggesting it might improve clinical pregnancy rate, the current data lacks high-quality RCTs, thus the role of vitamin D supplementation in the therapy of infertility remains obscure and calls for further research.

### 9.3. Vitamin D and PCOS

PCOS affects a significant portion of fertile women with a prevalence of around 10% worldwide [172]. Our understanding of its pathogenesis is incomplete, but it is believed to be a multifactorial disorder with typical “polycystic” ovaries on ultrasound, high androgen levels, and chronic anovulation as described by the Rotterdam criteria [173]. Hallmarks of the disease are insulin resistance regardless of bodyweight, low-grade chronic inflammation, an abnormally high LH/FSH ratio, and abnormal steroidogenesis with elevated anti-Müllerian hormone, estrogen, total free androgens, and decreased sex hormone binding globulin levels. These processes result in a complex vicious cycle leading to a typical clinical syndrome of anovulation, hirsutism, alopecia, acne, metabolic syndrome, and type 2 diabetes mellitus [34,173,174,175,176].

There is a high prevalence of vitamin D deficiency (40–80%) amongst PCOS patients, with especially high rates amongst patients suffering from insulin resistance and metabolic syndrome [177,178], while evidence also points to vitamin D being essential for steroidogenesis, which has led to several studies investigating its role as a potential disease-modifying therapy in PCOS [179,180,181]. Pal et al. described that 25(OH)D was an independent predictor of ovulation [182], while Lerchbaum et al. found a negative correlation between 25(OH)D levels and LH/FSH ratio [183]. One study described improved insulin sensitivity and decreased androgen profile as a result of vitamin D repletion that was unique to women with PCOS [184]. Another study by Jamilian et al. compared high vs. low dose supplementation favoring high doses [185]. Tóth et al. [186] in a prospective multicentric RCT involving 84 patients with vitamin D deficiency, found that vitamin D supplementation (30,000 IU/week for 12 weeks) improved ovarian morphology, ovulation probability, cycle length, and regularity, and decreased testosterone levels, while emphasizing that there were significant differences in response with different phenotypes. Participants with a high FSH/LH (>2) ratio responded better to repletion, while those with higher androstenedione levels demonstrated only marginal effects in regard to ovulation, despite improved levels of testosterone and cycle length. Meanwhile several meta-analyses also found a benefit of vitamin D supplementation, the exact beneficial effects, dosing, and indications remain controversial and require further large RCTs [187,188,189]. Nonetheless, current guidelines support the correction of vitamin D deficiency as the part of complex preconception nutritional care [34]. Thus, screening patients diagnosed with PCOS for vitamin D deficiency may be justified, and identified deficiencies should be corrected, as supplementation is inexpensive and may improve clinical symptoms and conception rates.

### 9.4. Vitamin D and Endometriosis

Like PCOS, endometriosis is a widespread problem amongst fertile-aged women, with a prevalence of 10%, which may vary widely between regions [190]. The disease carries an enormous economic burden, with the average cost of care and loss of productivity estimated to be around EUR 10,000/patient [191]. Endometriosis is defined by the presence of ectopic endometrial tissue, thought to be a result of retrograde menstruation and a favorable endocrine microenvironment, but the exact pathophysiology is yet to be fully elucidated. These ectopic foci behave similarly to the physiologic endometrium, taking part in the menstrual cycle, which in turn leads to widely varying symptoms based on their location [192]. The most common symptoms are dysmenorrhea, pelvic pain syndrome, dyspareunia, dysuria, and fatigue. Pelvic pain seems to at least in part be neuropathic in nature, supported by the fact that it may persist after surgical removal of the lesion, and is refractory to conventional treatment in 30% of cases. The diagnosis is based on direct laparoscopic visualization and histologic confirmation [192].

First-line medical treatment is based on hormone production suppression therapy by oral contraceptives, which results in atrophy of ectopic and physiologic endometrial tissue. GnRH agonists and aromatase inhibitors are second-line agents with considerable side-effects, such as bone loss, limiting their clinical utility [192,193,194]. Complementary treatment of pelvic pain consists of analgesics, anxiolytics, and antidepressants, combined with non-pharmacologic interventions. Surgical treatment should be considered in hormone-refractory pain, but achieving a pain-free state is not guaranteed with around 13% of the cases showing no improvement after surgery. 

Excision of the endometriomas is also associated with decreased follicular reserves, thus adversely affecting fertility [195,196]. Therefore, a significant portion of women have debilitating symptoms despite conservative and surgical interventions, warranting further research into potential modifiable risk factors and alternative therapeutic options. One such factor that has been the subject of research in recent years is vitamin D. Vitamin D deficiency has been suggested to be a risk factor for endometriosis, with some observational studies suggesting that 70–85% of patients have vitamin D deficiency [197,198]. The proposed role of vitamin D in endometriosis, like in many other conditions, is based on its role in immunomodulation and anti-inflammatory pathways. Namely, it has been shown to decrease CD44, Interleukin 8, prostaglandin, and matrix metalloproteinase 2 and 9 expression by endometrial tissue, which are all implicated in endometrial cell invasion [199]. One small RCT of 34 patients demonstrated a decrease in CD44 activity, measured from eutopic endometrial fluid, suggesting that vitamin D supplementation may decrease the invasiveness of endometrial cells [200]. In another medium-sized (60 patients) RCT with a 2-week follow up Mehdizadehkashi et al. described statistically significant improvements regarding pelvic pain, high-sensitivity CRP, total antioxidant capacity, and total-/HDL-cholesterol ratio, corroborating the postulated anti-inflammatory effects of vitamin D in endometriosis [201]. However, others failed to reproduce the previously mentioned positive effect on pelvic pain. The SAGE study compared omega-3 and vitamin D3 supplementation to placebo with a follow-up period of 6 months in 69 patients. They found no significant improvement in pelvic pain nor in quality of life in either intervention group [202]. Nevertheless, it is important to note that all patients involved were vitamin D sufficient, while in the study by Mehdizadehkashi et al. [201], all were at least insufficient, which might in part explain the discrepancy.

In conclusion, current evidence is insufficient to recommend vitamin D supplementation in endometriosis patients. Data are contradictory, and the field is understudied. Larger studies with an appropriate population size and comparison of vitamin D-sufficient and -deficient patients are required.

### 9.5. Vitamin D in Male Infertility

In males, vitamin D has been investigated for two major potential roles. Firstly, its effects on testosterone, SHBG, estradiol, amongst other sex hormones. The other major topic of interest was spermatogenesis and sperm function. In regard to steroidogenesis and sex hormone profiles, most observational and interventional studies do not support the link between testosterone (T) and vitamin D status, nor supplementation as an intervention to increase T levels [203,204]. In children with 1,25(OH)D3 resistant rickets, there was normal testosterone secretion to hCG stimulation, suggesting that it plays no essential role in testosterone secretion [203,205]. There is some evidence that vitamin D shows a positive correlation with SHBG levels and thus might influence the bioavailability of testosterone [203,206].

Vitamin D status seems to have little effect on estradiol levels in males. However, high-quality evidence in this field is lacking. The most crucial limitation identified by previous authors is the highly variable findings amongst available studies, which could be attributed to large differences in study designs and populations. A major limitation of available data is that only taking vitamin D status into account fails to address the many internal and external factors influencing sexual physiology, such as age, obesity, comorbidities, exposure to environmental toxicants, nutritional status, and other hormonal axes [203,204,207,208].

Vitamin D seems to be promising regarding sperm quality, specifically sperm motility. Two large meta-analyses both demonstrated a positive and significant correlation between 25(OH)D3 levels, sperm motility, and progressive motility, which are key factors in successful fertilization, while there were no statistically significant changes described in other sperm quality parameters such as sperm count or morphology [209,210]. The underlying mechanism has been attributed to the VDR-mediated effect by which vitamin D increases intracellular calcium levels through a nongenomic action [204,211]. On the contrary, a large RCT involving 330 men only demonstrated positive outcomes in the oligozoospermic and vitamin-deficient subgroup with improved livebirth rate, while there was no statistically significant improvement in sperm quality or live births despite improved 25(OH)D3 levels in the vitamin D group [212]. Another consistent result was that vitamin D levels show a positive correlation with fertility, with infertile men having considerably lower vitamin D levels [209,210,212]. While this finding suggests a link, it is important to state that these results are from observational and not interventional studies.

Based on current evidence, no recommendation can be given about vitamin D status monitoring and supplementation in infertile men. Further studies are required to clarify this new frontier of male reproductive endocrinology.

## 10. Conclusions

Vitamin D remains an inexpensive and safe intervention in several endocrinopathies such as osteomalacia, osteoporosis, and primary hyperparathyroidism. Screening for deficiency as part of preconception care in PCOS is supported by current guidelines. We summarized the current evidence below in Table 1. In conclusion, vitamin D’s pleiotropic effects are well established in in vitro and animal models. However, many of its suggested benefits are yet to be established in clinical trials. Our review highlights the need for well-designed large RCTs in several endocrine disorders where vitamin D’s role is still controversial.

## Figures and Tables

**Table 1:** Summary of current evidence and controversies [19,34,42,45,46,52,53,58,64,75,76,85,87,89,91,92,103,119,120,129,131,133,134,135,137,138,140,141,147,152,157,158,160,162,164,166,169,171,183,187,188,189,197,198,199,200,201,202].

Endocrine Disorder	Vitamin D Status	Established Role of Supplementation	Controversial/Potential Role	Key Evidence Summary
Osteoporosis	Deficiency is a major risk factor	Supported. Combined with calcium, for high-risk patients and those on anti-resorptive therapy	Optimal dosing and use in the general population remain debated	Meta-analyses support fracture reduction at 800–1000 IU/day, but some large RCTs (e.g., VITAL) found no benefit in the general population
Primary Hyperparathyroidism	Deficiency/insufficiency is highly prevalent (up to 81%)	Supported. Maintain levels of 30–50 ng/mL	The low 25(OH)D3 may be due to increased conversion to 1,25(OH)D3, not a “true” deficiency.	Studies show repletion is safe, lowers PTH, and improves the biochemical profile without worsening hypercalcemia.
Hypoparathyroidism	Unknown	Supported. Active vitamin D supplementation is the cornerstone of therapy, alongside calcium supplementation.	The exact benefit of non-active vitamin D supplementation is still debated	Guidelines favor active forms for precise titration. Non-active forms are used as an adjunct. A 25(OH)D3 level of 30–50 ng/mL is recommended.
Osteomalacia	Deficiency is the most common cause.	Supported. Causality is proven. Therapy is directed at the underlying deficiency.	There are no controversies	Diagnosis is often missed. Guidelines recommend loading doses followed by maintenance therapy.
Hashimoto’s Thyroiditis (HT)	High prevalence of deficiency An inverse correlation was found in many studies.	Not Supported.	May reduce ATPO/ATG levels and risk of progression, but evidence is inconsistent.	Observational studies are promising, but the field lacks large RCTs, and some studies show no association.
Postpartum Thyroiditis (PPT)	Limited data: pilot studies suggest a correlation with low levels.	Not Supported.	May have an additive benefit to L-thyroxine in lowering ATPO levels.	Evidence is limited to a few small observational and pilot studies.
Basedow–Graves’ Disease (BGD)	Associated with lower 25(OH)D3 levels.	Not Supported.	Observational data suggested a link, but interventional trials failed to show benefit.	Large trials (e.g., DAGMAR) found no benefit from supplementation in preventing recurrence or improving TRAb titers.
Thyroid Cancer	Association described in in vitro and animal models.	Not Supported.	In vitro data are compelling (e.g., reduces cell migration)	No RCTs are available.
Female Infertility	High prevalence of deficiency (27–80%) in infertile women.	Not Supported.	May improve clinical pregnancy rates, especially in deficient patients, but the evidence is contradictory	Meta-analyses show mixed results. The SUNDRO trial found no benefit for clinical pregnancy rates in IVF
Polycystic Ovary Syndrome	High prevalence of deficiency (40–80%)	Supported (deficiency only). Preconception nutritional care.	May improve ovulation, cycle regularity, and androgen levels	Several meta-analyses and RCTs found benefits, but the exact dosing and effects remain debated.
Endometriosis	High prevalence of deficiency (70–85%)	Not Supported.	May reduce pelvic pain and inflammation	The evidence is insufficient and contradictory. The SAGE trial found no improvement in pelvic pain in vitamin D-sufficient patients
Male Infertility	Infertile men often have lower vitamin D levels.	Not Supported.	Positively correlated with sperm motility.	No consistent evidence for improving testosterone levels. A large RCT found no improvement in sperm quality or live births in the general group.

## Data Availability

No new data were created or analyzed in this study. Data sharing is not applicable to this article.

## Abbreviations

The following abbreviations are used in this manuscript:

AMH	Anti-Müllerian hormone
ATG	Anti-thyroglobulin antibody
ATPO	Anti-thyroid peroxidase antibody
BGD	Basedow–Graves’ disease
BMD	Bone mineral density
CRP	C-reactive protein
DEXA	Dual-energy X-ray absorptiometry
FGF23	Fibroblast growth factor 23
FRAX	Fracture Risk Assessment Tool
FSH	Follicle-stimulating hormone
GnRH	Gonadotropin-releasing hormone
hCG	Human chorionic gonadotropin
HDL	High-density lipoprotein
HT	Hashimoto thyroiditis
IU	International Units
IVF	In vitro fertilization
LH	Luteinizing hormone
PCOS	Polycystic Ovary Syndrome
PHPT	Primary hyperparathyroidism
PPT	Painless postpartum thyroiditis
PTH	Parathyroid hormone
RCT	Randomized Controlled Trial
SHBG	Sex hormone binding globulin
T	Testosterone
TNF-alfa	Tumor necrosis factor alpha
TRAb	TSH receptor antibodies
TSH	Thyroid stimulating hormone
VDR	Vitamin D receptor
1,25(OH)2 D3	1,25-dihydroxyvitamin D3
25(OH)D3	25-hydroxyvitamin D3
